# The Relation of Insulin Resistance With Free Androgen Index and Endothelial Dysfunction Marker YKL-40 and Its Effect on Male Infertility: A Prospective Study

**DOI:** 10.7759/cureus.72481

**Published:** 2024-10-27

**Authors:** Muserref Banu Yilmaz, Betül Keyif

**Affiliations:** 1 Department of Obstetrics and Gynecology, SBU (Sağlık Bilimleri Üniversitesi) Zeynep Kamil Women and Children’s Diseases Training and Research Hospital, İstanbul, TUR; 2 Department of Obstetrics and Gynecology, Düzce University, Düzce, TUR

**Keywords:** endothelial dysfunction, free androgen index, insulin resistance, male infertility, ykl-40

## Abstract

Purpose: In this study, the effects of insulin resistance and the role of the endothelial dysfunction marker YKL-40 in male infertility were investigated.

Methods: This study was conducted with male patients who underwent fertility investigations in the in vitro fertilization (IVF) unit of a tertiary center. Semen samples were evaluated after two to five days of sexual abstinence. The hormonal and lipid profiles, fasting glucose and insulin levels, and YKL-40 values of the patients were assessed. The Homeostatic Model Assessment (HOMA) index was calculated, and those with a score of 2.5 and above were considered insulin resistant (IR).

Results: In total, 75 patients, 34 with IR and 41 without IR, were included. The groups had similar ages, heights, weights, and BMIs. Additionally, the semen parameters were similar between the groups. The median fasting glucose and insulin levels were significantly greater in the insulin-resistant group (p=0.008 and p<0.00, respectively). There were no significant differences in terms of the serum follicle-stimulating hormone (FSH), luteinizing hormone (LH), prolactin (PRL), thyroid stimulating hormone (TSH), sex hormone binding globulin (SHBG), high-density lipoprotein (HDL), or low-density lipoprotein LDL) levels. Total testosterone levels were lower in the IR group than in the group without IR (p<0.001). Both the total serum cholesterol and triglyceride levels were greater in the IR patients (p=0.031 and p=0,002, respectively). Compared with the patients without IR, the IR patients had a lower free androgen index. The serum YKL-40 levels were similar between the groups, and there were no correlations between YKL-40 and the other parameters.

Conclusion: This initial study investigating the serum YKL-40 level in men with and without IR revealed no relationship between YKL-40 levels and IR. Nonetheless, higher total serum cholesterol and triglyceride levels and lower total serum testosterone and free androgen indices were found in participants with IR.

## Introduction

The quality of semen is influenced by many factors, ranging from genetics to diseases based on lifestyle. Given the increased prevalence of obesity and metabolic disturbances, researchers have focused on the effects of glucose and lipid metabolism on spermatogenesis to reveal the relationship between metabolic syndrome and male infertility [1,2]. While the role of insulin resistance (IR) in female fertility has been widely studied, the influence of IR on male fertility has not yet been clearly identified. It has been hypothesized that IR among men is often connected with metabolic syndrome and obesity and is associated with decreased sperm quality [3].

YKL-40 is a chitinase-like protein that is expressed by chondrocytes, neutrophils, macrophages, vascular smooth muscle cells, fibroblast-like synovial cells, and hepatic stellate cells. YKL-40 may play a role in many pathogenic processes, including rheumatoid arthritis, osteoarthritis, giant cell arteritis, sarcoidosis, liver fibrosis, and malignant diseases, via inflammation, extracellular tissue remodeling, and the regulation of cell-matrix interactions [4,5]. YKL-40 is also thought to be a marker of endothelial dysfunction in metabolic disturbances such as diabetes mellitus [6]. In a study on the effect of metabolic syndrome on male infertility, YKL-40 was presented as a reliable marker [4], but the mechanism of infertility and the relationship between YKL-40 and IR have not been clarified.

Wilson et al. reported increased plasma YKL-40 levels and a positive correlation between plasma YKL-40 levels and Homeostatic Model Assessment (HOMA)-IR in patients diagnosed with type 2 diabetes [7]. In another study, Hanafy et al. reported higher serum levels of YKL-40 in male patients diagnosed with idiopathic infertility than in fertile participants [4]. Thus, we thought that there may be a relationship between semen parameters, reproductive hormones, HOMA-IR, and serum YKL-40 levels.

For this purpose, we investigated the effect of IR on male fertility and the role of YKL-40 as a marker of endothelial dysfunction with and without IR.

## Materials and methods

This was a prospective study conducted at the Assisted Reproduction Unit of Zeynep Kamil Women and Children’s Diseases Training and Research Hospital, Istanbul, Türkiye, between October 2020 and January 2021. A total of 75 male patients, aged 25-55 years, who presented for fertility investigation were enrolled in the study. The study was approved by the Research Ethics Committee of the SBU (Sağlık Bilimleri Üniversitesi) Zeynep Kamil Women and Children’s Disease Training and Research Hospital (approval number: 98/2019). All participants provided written informed consent before their inclusion in the study, and the study was conducted in accordance with the Declaration of Helsinki. 

Selection of participants

Inclusion criteria were male patients between 25 and 55 years of age who underwent fertility evaluation and provided informed consent for participation in the study. Patients were excluded if they had any chronic diseases such as diabetes mellitus, hypertension, azoospermia, varicocele, or malignancies, or were involved in substance abuse (smoking or alcohol consumption). Additionally, patients with known cardiovascular diseases, inflammatory conditions, or those taking medications that could affect reproductive hormones were also excluded.

A convenience sampling method was employed for patient recruitment. All eligible patients presenting to the unit during the study period were approached, and those who met the inclusion criteria and agreed to participate were included in the study.

Data collection and laboratory assessments

Demographic data, including age, weight, height, and body mass index (BMI), were recorded at the initial visit. Blood samples were collected after an 8-10 hour fasting period at 9:00 am and centrifuged immediately. Serum total testosterone (TT), sex hormone-binding globulin (SHBG), prolactin (PRL), follicle-stimulating hormone (FSH), luteinizing hormone (LH), thyroid-stimulating hormone (TSH), fasting insulin (FI), fasting glucose (FG), YKL-40 levels, and serum lipid profiles were evaluated on the same day at the biochemistry laboratories of the SBU Zeynep Kamil Women and Children’s Disease Training and Research Hospital. YKL-40 levels are measured by using a human chitinase-3-like protein 1 (YKL-40/CHI3L1) enzyme-linked immunosorbent assay (ELISA) kit which was based on the principle of double-antibody sandwich technique (Shanghai Sunred Biological Technology Co., China).

IR Assessment

HOMA indexes were calculated with FG (mmol/L) and FI (μIU/mL) according to the following formula:

$\frac{FG\times FI\times 0.055}{22.5}$ [8]. 

Patients with a HOMA-IR score of ≥ 2.8 were classified as insulin resistant, while those with a HOMA-IR score < 2.8 were classified as non-insulin resistant [9]. Two groups were formed based on the presence or absence of IR and semen parameters, hormonal values, and metabolic parameters were compared between the two groups.

Semen Analysis

Semen samples were obtained after two to five days of sexual abstinence and evaluated according to the guidelines given in the WHO Laboratory Manual for the Examination and Processing of Human Semen (5th edition) [10]. Parameters such as sperm concentration, total sperm motility, and total motile sperm count were recorded.

Statistical analysis

Data were analyzed using the IBM SPSS Statistics for Windows, Version 20.0 (Released 2011; IBM Corp., Armonk, New York, United States). Descriptive statistics, including mean ± standard deviation (SD), median, and ranges, were used to describe continuous variables. Categorical data were expressed as numbers and percentages, while quantitative data were expressed as median ± SD and range. The demographic, laboratory, and semen parameters in the two groups were compared using an independent sample t-test. Pearson correlation analysis was used to evaluate the relationships between YKL-40 levels and other clinical parameters. A p-value of less than 0.05 was considered statistically significant.

## Results

This study included 75 male patients, 34 of whom were insulin resistant and 41 of whom were not insulin resistant. The two groups had similar mean age, height, weight, BMI, and waist circumference values. Analysis revealed that the median fasting glucose and insulin levels were significantly greater in the group with IR (p=0.008 and p<0.001, respectively). Patients with IR had higher HOMA indices than patients without IR (p<0.001) (Table 1).

**Table 1:** Characteristics of the participants with and without insulin resistance *p<0.05. HOMA-IR: Homeostatic Model Assessment for Insulin Resistance; IR: insulin resistance

	Participants with IR (n=34), mean±SD	Participants without IR (n=41), mean±SD			P value
Age (years)	35.41 ± 4.61	34.78 ± 5.52			0.591
Weight (cm)	84.38 ± 4.47	82.41 ± 10.45			0.346
Height (kg)	175.09 ± 5.47	176.05 ± 6.32			0.483
BMI (kg/m^2^)	27.53 ± 2.18	26.56 ± 2.82			0.100
Waist circumference (cm)	94.00 ± 10.72	91.68 ± 11.79			0.376
Fasting blood glucose (mg/dl)	96.47 ± 9.32	90.93 ± 8.11			0.008*
Fasting serum insulin (mIU/ml)	34.07 ± 21.94	7.93 ± 2.46			0.000*
HOMA-IR	8.07 ± 5.44	1.76 ± 0.56			0.000*

Semen parameters, including sperm concentration, total sperm motility, and total motile sperm count, were similar between both sets of participants (Table 2).

**Table 2:** Semen parameters of the participants with and without insulin resistance *p<0.05 IR: insulin resistance

Semen parameters	Participants with IR (n=34), mean±SD	Participants without IR (n=41), mean±SD	P value
Sperm concentration (million/mL)	46.63 ± 35.16	47.30 ± 37.16	0.937
Total sperm motility (%)	49.41 ± 20.34	48.73 ± 21.76	0.889
Total motile sperm count (x10^6^)	73.50 ± 82.36	78.07 ± 72.29	0.802

There were no significant differences in terms of serum FSH, LH, PRL, TSH, SHBG, HDL, or LDL levels. Total testosterone levels were lower in the group with IR than in the group without IR (p<0.001). Both total serum cholesterol and triglyceride levels were greater in insulin-resistant patients (p=0.031 and p=0,002, respectively). Patients with IR had lower free androgen index values than the patients without IR. The serum YKL-40 levels were similar between the groups (Table 3). 

**Table 3:** Hormonal and lipid profiles of the participants *p<0.05. FSH: follicle-stimulating hormone; LH: luteinizing hormone; SHBG: sex hormone-binding globulin; PRL: prolactin; TSH: thyroid-stimulating hormone; YKL-40: chitinase-like protein; IR: insulin resistance

	Participants with IR (n=34), mean±SD	Participants with IR (n=41), mean±SD	P value
FSH (mIU/ml)	4.20±3.05	3.72±2.09	0.441
LH (mIU/ml)	5.26±11.26	3.21±1.66	0.256
Prolactin (ng/ml)	11.15±4.97	10.85±6.60	0.823
TSH (mU/L)	1.61±0.74	1.27±0.53	0.027
Total testosterone (ng/ml)	3.49±1.23	5.16±2.10	0.000*
SHBG (nmol/L)	20.42±6.64	24.02±8.89	0.055
Free Androgen Index	16.63±8.07	27.52±14.33	0.000*
Total cholesterol (mg/dl)	198.97±52.36	176.10±32.61	0.031*
Triglycerides (mg/dl)	189.18±93.72	130.56±57.06	0.002*
High-density lipoproteins (mg/dl)	39.00±27.67	41.68±15.50	0.617
Low-density lipoproteins (mg/dl)	124.53±51.30	105.29±30.91	0.061
Very-low-density lipoproteins (mg/dl)	37.83±18.74	26.11±11.59	0.002*
YKL40 (ng/ml)	77.02±73.89	75.36±69.94	0.921

Correlation analysis revealed no correlations between serum levels of YKL-40 and the other parameters (Table 4).

**Table 4:** Correlations between YKL-40 and clinical laboratory data HOMA-IR: Homeostatic Model Assessment for Insulin Resistance; SHBG: sex hormone-binding globulin

Parameters	r	P value
Sperm concentration	0.216	0.063
Total sperm motility	0.113	0.336
Total motile sperm count	0.184	0.113
Total testosterone	0.062	0.599
SHBG	0.227	0.050
Free androgen index	0.944	0.503
HOMA-IR	-0.138	0.236
Fasting serum insulin	0.062	0.189
Fasting blood glucose	0.132	0.258
BMI	-0.008	0.944

## Discussion

In this study, in connection with the knowledge of the detrimental effects of metabolic disturbances such as diabetes mellitus on male fertility [6], IR and YKL-40, which are inflammatory markers, were hypothesized to be related to decreased semen quality due to exaggerated insulin secretion, impaired glucose metabolism, and inflammation. The present study reported higher total serum cholesterol and triglyceride levels and lower total serum testosterone and free androgen index values in male patients with IR than in those without IR. Additionally, both groups had similar YKL-40 values.

Infertility is a serious problem, and the number of infertile couples increases daily. Problems such as poor sleep quality, poor diet, physical activity, or metabolic disorders affecting spermatogenesis or spermiogenesis can cause male infertility. Diabetes occurs in cases of untreated IR, and these patients require more insulin to transport glucose. Additionally, IR leads to inflammation in body cells, which negatively affects the quality of semen [8,9]. In the present study, lower total serum testosterone and free androgen index values were detected in participants with IR. Consistent with our results, Lotti et al. reported reduced total testosterone levels in men with type 1 diabetes as well as type 2 diabetes [10]. IR in cells leads to a lack of insulin stimulation of Leydig cells, and this defect causes a reduction in testosterone production [11].

YKL-40, a reliable marker of endothelial dysfunction, is expressed by neutrophils and macrophages and plays a major role in inflammation and angiogenesis [12,13]. In a study by Hanafy et al. investigating serum YKL-40 levels in male patients diagnosed with idiopathic infertility, they measured serum YKL-40 levels in patients with varicocele to evaluate the role of endothelial dysfunction in these patients and reported higher serum YKL40 levels in these patients as compared to the controls [14]. In another study investigating the serum levels of YKL-40 in preeclampsia patients, Seol et al. reported elevated serum YKL-40 in patients with preeclampsia compared with controls, and these increases were related to the severity of the disease [15]. In a recent study, Tuten et al. reported significantly higher HOMA-IR values and serum YKL-40 levels in pregnant women with diabetes than in controls [16]. Additionally, they reported a positive correlation between these two parameters. Inconsistent with the results of these studies [6,14], we observed similar serum YKL-40 levels in male patients with IR compared to patients without IR. We also observed no correlation between serum YKL-40 and other parameters, including HOMA-IR, semen parameters, and reproductive hormones. This discrepancy between our findings and those of the above studies might be due to differences in the studied population and the duration of IR.

This is the first study to investigate the serum level of YKL-40, a marker of endothelial damage in male patients with and without IR, and the relationships of this marker with semen parameters and reproductive hormones. A limitation of our study is the lack of long-term results, including the fertility status of the participants. More comprehensive studies are needed, including the results of infertility treatments in male patients with IR.

## Conclusions

This initial study investigating the serum YKL-40 level in male patients with and without IR revealed no relationship between YKL-40 levels and IR. Nonetheless, higher total serum cholesterol and triglyceride levels and lower total serum testosterone and free androgen indices were found in IR males. These findings suggest that IR has a more pronounced effect on lipid metabolism and reproductive hormones in males, potentially contributing to fertility issues. Further long-term studies with larger sample sizes are needed to confirm these findings and explore the potential role of YKL-40 in male reproductive health. Additionally, investigating the underlying mechanisms of IR on male fertility could provide new insights for therapeutic approaches. 
